# Pancreaticoduodenal Artery Aneurysm Associated with Celiac Trunk Stenosis: Case Illustration and Literature Review

**DOI:** 10.1155/2017/6989673

**Published:** 2017-07-26

**Authors:** Jad A. Degheili, Alissar El Chediak, Mohamad Yasser R. Dergham, Aghiad Al-Kutoubi, Ali H. Hallal

**Affiliations:** ^1^Department of Surgery, Division of General Surgery, American University of Beirut Medical Center, Beirut, Lebanon; ^2^Department of Internal Medicine, American University of Beirut Medical Center, Beirut, Lebanon; ^3^Department of Diagnostic Radiology, Division of Interventional Radiology, American University of Beirut Medical Center, Beirut, Lebanon

## Abstract

Pancreaticoduodenal artery aneurysms (PDA) are rare visceral aneurysms. Celiac trunk stenosis represents a common attributable aetiology for those aneurysms. Therefore, an alternative treatment approach, which differs from those isolated aneurysms, is recommended. We hereby present a 77-year-old male patient who was admitted with sudden onset of severe abdominal pain and significant drop in haemoglobin, occurring within a 24-hour interval. Contrast-enhanced computed tomography revealed a ruptured visceral aneurysm arising from the anterior branch of the inferior pancreaticoduodenal artery. A severe stenosis was also noted at the take-off of the celiac trunk. Selective catheterization of the supplying branch of the superior mesenteric artery, followed by coil embolization of the aneurysm, was performed, resulting in cessation of flow within the aneurysm, with preservation of the posterior branch, supplying the celiac territory. PDAs are usually asymptomatic and discovered incidentally at rupture. The risk of rupture is independent of the aneurysmal size and is associated with a 50% mortality rate. The consensus on coping with aneurysms is to treat them whenever they are discovered. Selective angiography followed by coil embolization represents a less invasive, and frequently definitive, approach than surgery. The risk for ischemia mandates that the celiac territory must not be compromised after embolization.

## 1. Background

Pancreaticoduodenal artery (PDA) aneurysms constitute around 2% of all splanchnic aneurysms [1]. Unlike other aneurysms, the risk of rupture is independent of the aneurysmal diameter. True PDA aneurysms have been linked to hemodynamic alterations in the involved branches caused by altered blood flow due to celiac trunk stenosis [2]. Enlargement of the pancreaticoduodenal arcade is also a contributing factor in the formation of aneurysms secondary to local inflammatory processes such as pancreatitis [3]. Increasing numbers of such cases are being reported due to increased use of cross sectional imaging, and a physiological association between PDA aneurysms and celiac trunk stenosis has been described. However, no unified treatment algorithm has been reported [4]. Transarterial embolization (TAE) is an effective and minimally invasive initial approach [5]. We present a case of a ruptured PDA aneurysm associated with celiac trunk stenosis that was successfully treated with transarterial coil embolization. In this case study, we highlight and discuss the association between celiac trunk stenosis and PDA aneurysms and discuss the various management options.

## 2. Case Description

**Figure 1:**
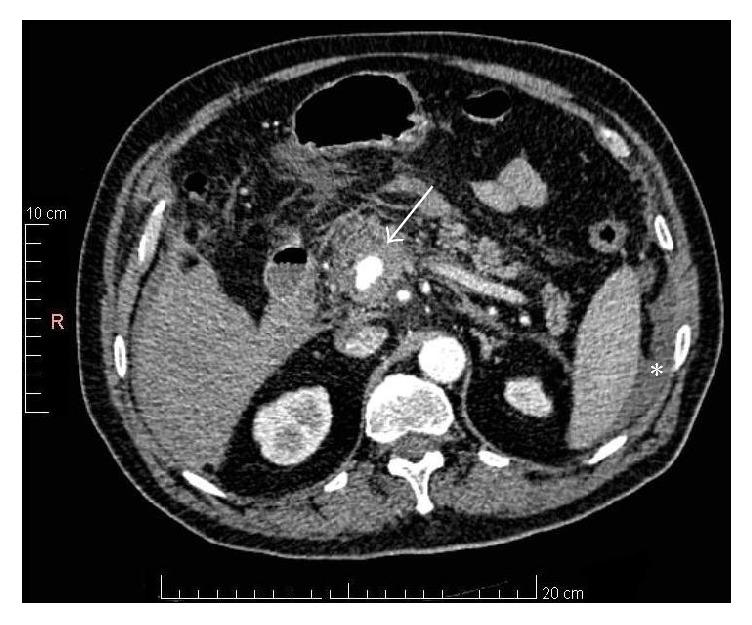
*Contrast-enhanced CT scan of the abdomen prior to transarterial embolization*: A 2.0- × 1.4 cm visceral aneurysm* (Arrow)* surrounded by 0.7 cm rim of thrombus arising from the inferior pancreaticoduodenal branch. The hemoperitoneum is also noticed in this figure* (Asterisk)*.

**Figure 2:**
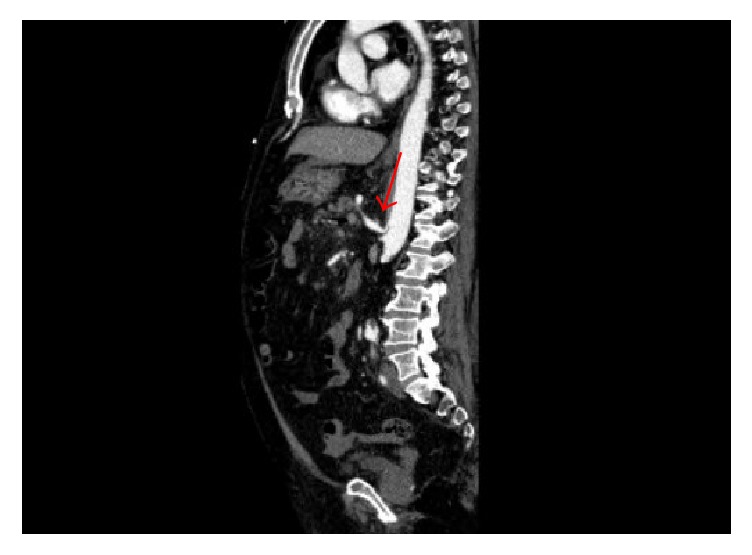
*Evidence of celiac trunk stenosis*: Sagittal reconstruction of contrast-enhanced CT scan of abdomen revealing the evidence of stenosis at the take-off of the celiac trunk (Arrow).

**Figure 3:**
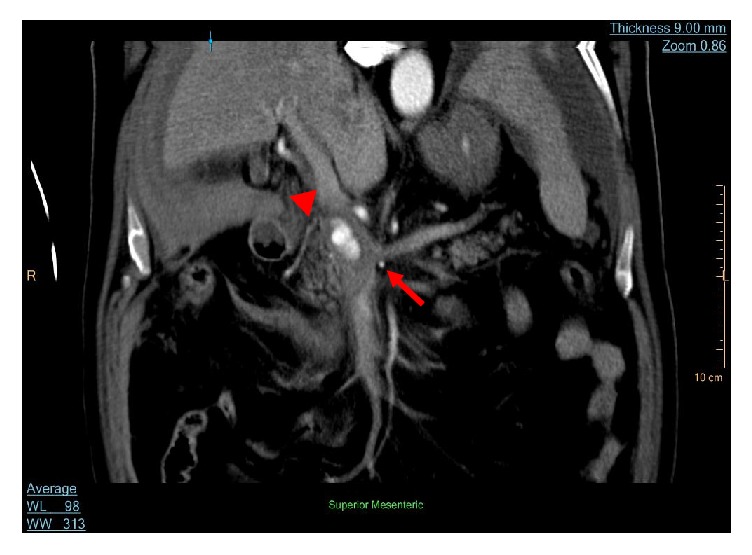
*Coronal reconstruction revealing patent portal vein (Arrowhead) with compression of confluence by the aneurysm*: filling of the aneurysm with contrast is shown with its respective compression on the confluence of the superior mesenteric vein and the portal vein* (Arrow)*.

A 77-year-old male resident of a nursing home, on antihypertensive and prophylactic anticoagulation medications, sustained a spontaneous postsyncopal head injury that resulted in subarachnoid haemorrhage. He was admitted to our hospital for close observation. One day later, he developed tachycardia with a heart rate reaching 120 bpm, hypotension with a systolic blood pressure of 80 mmHg, and an increase in abdominal girth, distention, and pain. His haemoglobin level decreased from 13.5 to 9.4 g/dL (reference range, 13–18 g/dL). His white blood cell count was 9800/mm^3^ (reference range, 4000–11,000/mm^3^), and his serum amylase level was 129 IU/L (reference range, 10–120 IU/L). Contrast-enhanced computed tomography (CT) of the abdomen and pelvis showed a 2.0- × 1.4-cm visceral aneurysm, surrounded by a 0.7 cm rim of thrombus, arising from the inferior pancreaticoduodenal branch of the superior mesenteric artery (SMA), in addition to a moderate hemoperitoneum (Figure 1). The aneurysm was associated with severe celiac trunk stenosis (Figure 2). The compression by the aneurysm caused almost complete occlusion of the confluence of the splenic and superior mesenteric veins; however, the portal vein was widely patent (Figure 3). After stabilization with blood and blood product components, selective transarterial embolization was performed.

The right femoral artery was accessed, and a 5-Fr sheath was placed. A 5-Fr SIM2 catheter (Terumo Interventional Systems, Somerset, NJ, USA) was advanced over a 150-cm, 0.035-in Terumo hydrophilic guide wire, and selective catheterization of the inferior pancreaticoduodenal artery from the superior mesenteric artery was performed. Retrograde flow to the common hepatic branch, all the way to the celiac trunk, was demonstrated through the pancreaticoduodenal arcade.

**Figure 4:**
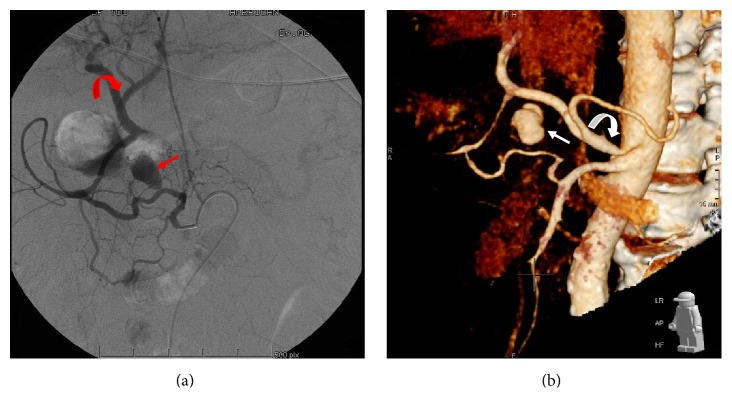
*Selective digital subtraction angiography (DSA) of the superior mesenteric artery*: (a) A 2.0- × 1.4-cm narrow neck oblong aneurysm* (Arrow)* originating from the anterior branch of the inferior pancreaticoduodenal artery. Note the several collaterals originating from the superior mesenteric artery and the retrograde filling of the hepatic artery* (Curved arrow)* through the pancreaticoduodenal arcade. (b) Three-dimensional reconstruction of the enhanced CT images revealing the oblong aneurysm* (Arrow)*, originating from a branch of the superior mesenteric artery. Note the stenosis at the origin of the celiac trunk* (Curved arrow).*

Filling of a single visceral aneurysm originating from an anterior branch of the Inferior PDA (Figures 4(a) and 4(b)) was observed. The aneurysm had an oblong shape arising from the superior aspect of the artery with a very narrow, almost pinpoint neck typical of a false aneurysm. There was also good flow through the posterior branch of the Inferior PDA towards the gastroduodenal arcade and the hepatic artery.

**Figure 5:**
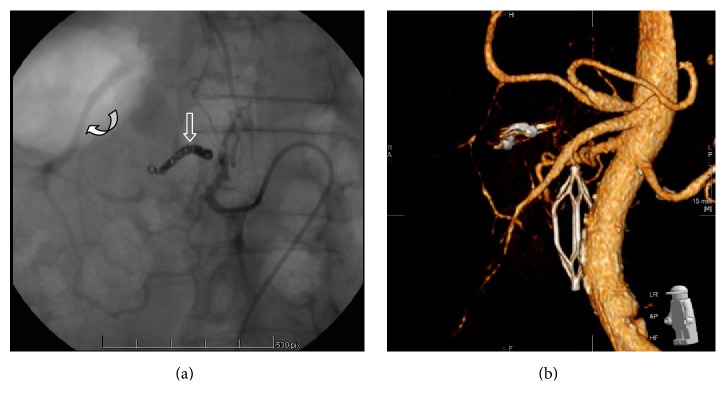
*Selective angiography after coil embolization*: (a) Microcoils* (Arrow)* proximal and distal to the origin of the aneurysm. Adequate retrograde collaterals to the liver through the posterior branch are also shown* (Curved arrow)*. (b) Three-dimensional reconstruction on follow-up CT scan, showing persistent occlusion of the aneurysm, with patent hepatic artery.

The close proximity of the aneurysm to the pancreas and SMV precluded percutaneous thrombin injection. The parent artery was measured at 4 mm in diameter and was then embolized by advancing a 2.8-F Prograte microcatheter (Terumo Interventional Systems) beyond the neck of the aneurysm and the placement of five interlocking 4 mm fibred platinum microcoils (Cook, Bloomingdale, USA), using the “back door-front door” approach, distal, across, and proximal to the origin of the aneurysm (Figure 5(a)). Three millilitres of Gelfoam (Pharmacia and Upjohn Co., Kalamazoo, MI, USA) was introduced into the coiled segment to further promote thrombosis. The posterior branch of the Inferior PDA remained patent and supplying the gastroduodenal arcade and the hepatic artery.

Completion angiography of the SMA revealed cessation of the flow into the aneurysm. The patient's hemodynamic parameters and clinical condition were then stabilized with blood product resuscitation.

## 3. Outcome and Follow-Up

The patient remained hemodynamically stable, with an almost constant haemoglobin level at 10.4 g/dL and normal liver function test results. His hospital course was protracted with requirement of drainage of ascites and IVC filter insertion. CT angiography performed 5 weeks after embolization for suspicion of bowel ischemia revealed thrombosis of the false aneurysm and a patent hepatic artery and portal vein, with no evidence of liver ischemia (Figure 5(b))

The patient's condition later deteriorated, and he developed severe pneumonia, with acute kidney injury necessitating supportive intubation and initiation of sepsis protocols. He died 40 days after his initial embolization.

## 4. Discussion and Evaluation

Splanchnic aneurysms can be classified based on their anatomical locations [6]. Visceral aneurysms are the least prevalent among all systematic aneurysms [7]. Several aetiologies have been correlated with their pathogenesis. Infection, trauma from surgeries or endoscopic procedures, collagen diseases, and pancreatitis [8] form the basis for the formation of pseudoaneurysms which becomes more prevalent when the arcade is enlarged secondary to celiac trunk stenosis [9]. True aneurysms have been linked to flow redistribution from celiac trunk stenosis [9]. The high flow rate or kinetics of turbulent blood in the smaller branches of the SMA, as a result of divergent blood from the celiac trunk due to stenosis, increases the shear stress on the single endothelial layer of the intima, resulting in alteration of its biochemical profile, the development of erosion, and increased permeability. These changes reflect deeply into the media layer. The media layer, which maintains the integrity and elasticity of vessels, became itself dysfunctional, resulting in aneurysmal formation [10]. True aneurysms are recognized in 0.09% to 2.00% of the general population [11].

### 4.1. Celiac Trunk Stenosis and PDA Aneurysms

The pathogenesis behind celiac trunk stenosis may be intrinsic in nature (e.g., caused by atherosclerosis or dysplasia) or extrinsic (e.g., caused by median arcuate ligament compression, which is seen in 10–24% of patients with celiac stenosis) [4]. The first reported case of a PDA aneurysm was published by Ferguson [12]. More than 131 cases of PDA aneurysms have been reported to date; 81 of which were linked to celiac trunk stenosis or occlusion [2]. The initial reported case that correlated PDA aneurysms with celiac trunk occlusion was described by Sutton and Lawton [13]. Since then, the reported incidence has ranged from 45% to 67% [1]. Accumulating evidence reveals that 50% to 80% of PDA aneurysms are associated with celiac artery stenosis [14]. De Perrot et al. [15] reported that 63% of true PDA aneurysms were associated with celiac trunk stenosis. In most reported cases, the cause of this stenosis is ambiguous. Of 12 reported cases with a known aetiology, 9 were attributed to median arcuate ligament compression and 3 were due to atherosclerosis, thrombosis, and agenesis of the celiac trunk, respectively [9].

### 4.2. Detection of PDA Aneurysms

With the advent and increased utility of imaging techniques, the detection of PDA aneurysms is becoming more frequently reported in the literature. Modalities used for detection include contrast-enhanced multidetector-row CT, three-dimensional contrast-enhanced magnetic resonance angiography [11], and CT angiography with 3D reconstruction from thin section (0.6 mm–3 mm) acquisitions [9, 16]. In addition, CT angiography and magnetic resonance angiography are capable of routinely detecting aneurysms less than 1 cm in diameter [17]. More recently, flow-sensitive four-dimensional magnetic resonance imaging has been implemented in studying chronic hyperkinetic flow in the pancreaticoduodenal arcade secondary to blood shifting from the celiac trunk to the SMA branches in the presence of stenosis. This hyperkinetic flow has been shown to form the basis for the formation of PDA aneurysms [14]. Despite these noninvasive modalities, selective digital subtraction angiography remains the gold standard for the diagnosis of PDA aneurysms because the location of the aneurysm and the supplying artery can be determined, and definitive treatment can be simultaneously performed through TAE.

With the presence of celiac trunk stenosis and the consequent divergent of retrograde blood from the SMA to the celiac territory, the arcade becomes engorged and easily visualized by SMA angiography and MRI kinetic studies [9, 14].

### 4.3. Management

No treatment guidelines have been established for the management of PDA aneurysms. Consensus states that such aneurysms must be treated, once detected. With a gastrointestinal haemorrhage incidence of 7% to 15% [18], mostly into the retroperitoneal cavity [19], the presence of PDA aneurysms is considered life-threatening. No correlation exists between the size of the PDA aneurysm and the rate of rupture; however, rupture is associated with a significant mortality rate reaching 50% [4, 5, 9] or even higher (up to 75%) [11]. Approximately 17.6% of ruptured aneurysms are ≤10 mm in diameter [1]. Suzuki et al. [20] reported a similar mean diameter (22.2 versus 21.4 mm) among PDA aneurysms that did and did not rupture, respectively. These facts render PDA aneurysms unique with respect to other visceral aneurysms, thus necessitating rigorous planning and implementation of treatment upon recognition.

A major goal in the treatment of aneurysms associated with celiac trunk stenosis revolves around obliteration, resolution of any associated pathologies, and maintenance of adequate blood flow to territories of the celiac trunk. Surgical options oscillate between ligation/resection and aneurysmorrhaphy. These treatments are associated with a high mortality rate and technical difficulties, especially after rupture. Continuous monitoring of the hepatic venous saturation through a right hepatic venous catheter could act as a surrogate approach to ensure an adequate blood supply to the celiac territories after resection or ligation of PDA aneurysms [21]. A saturation level of >60% indicates adequate hepatic perfusion [21].

Less invasive techniques, including TAE with or without recanalization or bypass of celiac stenosis, have recently been predominating. Celiac trunk recanalization promotes stagnation of blood within the aneurysm, resulting in regression in the size of the aneurysm by formation of an intramural thrombus [1]. No cases of PDA aneurysm recurrence after successful endovascular embolization alone have been reported, even without the resumption of adequate celiac flow. Considering this evidence, Suzuki et al. [5] stated that if the ischemic risk to the liver and duodenum is not significant, there is no need to reverse the stenosis. In such an approach, monitoring any ischemic insult to the liver would include serial monitoring of liver function tests, after embolization.

Transcatheter embolization may take the form of total occlusion of the parent artery in cases of fusiform aneurysms, or coil embolization of the aneurysm itself if it is saccular and its neck is accessible. Occlusion of the parent artery beyond and proximal to the neck of the aneurysm (back door/front door technique) is mandatory to prevent retrograde filling. However it is not always successful because vessels can be tortuous and difficult to bypass for deployment of embolic agents. However the development of new trackable microcatheters has improved the ability of the interventional radiologist to reach the target vessel and optimize the embolization procedure. Recurrence of an aneurysm after embolization may occur because collaterals can be excessive and difficult to access and occlude completely [22]. Alternative techniques such as percutaneous thrombin injection (PTI) under CT or ultrasound guidance have been implemented for the treatment of false aneurysms, thus providing patients with more options for minimally invasive procedures before proceeding to surgery [23]. PTI was pioneered by Cope and Zeit in 1986 [24] and since then has shown success in obliterating the aneurysmal sac through thrombin injection and thrombus formation without the need to embolize inflow and outflow vessels. With the use of 21-gauge or smaller calibre needles for percutaneous access, the risk of major organ-specific complications ranges from 0.1% to 2.0% [23]. PTI is characterized by a shorter overall procedure time and lower operational cost than transarterial embolization [22]. We did not attempt this approach because of the patient's moderate hemoperitoneum, fear of bowel perforation, and the proximity of the aneurysmal sac to pivotal organs, as well as the absence of real-time evaluation of the amount of thrombin being administered using CT guidance [23], all of which rendered this approach unfavourable.

The most concerning complication after TAE is reperfusion with subsequent rupture and bleeding into the abdominal cavity. The incidence of this complication depends on the technical success of the procedure and reportedly ranges from 5% to 20% [25], thus necessitating strict radiological follow-up. Some authors suggest imaging initially at 6 months and possibly yearly thereafter [17]. Further complications of embolization include ischemic injury to the liver, pancreas, or duodenum; tissue necrosis with subsequent abscess formation; and possible sepsis [26].

Several reports have described variations in the treatment of PDA aneurysms in conjunction with celiac trunk stenosis. Simultaneous treatment of the stenosis in conjunction with the aneurysm is still a matter of debate. Stambo et al. [27], Lossing et al. [28], and Savastano et al. [29] reported no recurrence of PDA aneurysms after transcatheter embolization and no need for revascularization of the celiac trunk. Others have reported complete obliteration of PDA aneurysms by formation of an intramural thrombus secondary to blood stagnation; only revascularization of the celiac trunk was performed in their situation, without resection or ligation of the aneurysm [1]. A contradictory report published by Suzuki et al. [20] negated the successfulness of embolization alone, without revascularization.

We did not attempt simultaneous celiac trunk recanalization as we considered this additional procedure an extra stress to our already hemodynamically unstable patient. The major concern would be to stop aneurysmal bleeding, at time of presentation. Had it been that our patient was stable enough, celiac trunk recanalization could be considered simultaneously, albeit the different contradictory, yet valid, viewpoints discussed in the pertinent literature.

In addition, Takao et al. [30] had reported the largest series of unruptured true pancreaticoduodenal artery aneurysms, followed without any intervention. Of the five reported patients with a total of eight true PDA aneurysms, four were associated with celiac trunk stenosis. Over a mean follow-up period of 29.4 months, three aneurysms increased in size, but no ruptured aneurysm occurred. Thus, in spite of the small sample size, the authors concluded that the risk of rupture of PDA aneurysms might be lower than predicted from other ruptured aneurysms' series.

Albeit TAE is not a novel approach for the treatment of ruptured PDA aneurysms associated with celiac trunk stenosis, it has been based only on single case series. The present descriptive case, along with the aforementioned literature review, has shed more light on this controversial topic.

## 5. Conclusion

PDA aneurysms are becoming more frequently reported in association with celiac trunk stenosis. The high mortality rate upon rupture necessitates abrupt treatment. No consensus exists regarding the optimal approach for treatment of these associated conditions. However, TAE is now accepted as an appropriate initial modality. The presence of celiac stenosis complicates the decision-making but is unlikely to require treatment. Whatever approach is used, maintenance of adequate perfusion to the celiac territory is pivotal. The performance of embolization, PTI, or surgery must be decided on a case-by-case basis until a well-established algorithm is devised.

## Abbreviations

PDA: Pancreaticoduodenal artery
IPDA: Inferior pancreaticoduodenal artery
TAE: Transarterial embolization
SMA: Superior mesenteric artery
PTI: Percutaneous thrombin injection.
